# Editorial: Shade avoidance syndrome in plants

**DOI:** 10.3389/fpls.2022.990982

**Published:** 2022-08-11

**Authors:** Lin Li, Haiyang Wang

**Affiliations:** ^1^State Key Laboratory of Genetic Engineering and Institute of Plant Biology, Institute of Plants Biology, School of Life Sciences, Fudan University, Shanghai, China; ^2^State Key Laboratory for Conservation and Utilization of Subtropical Agro-Bioresources, South China Agricultural University, Guangzhou, China; ^3^Guangdong Laboratory for Lingnan Modern Agriculture, Guangzhou, China

**Keywords:** shade avoidance, leaf senescence, nitrogen, soybean, phytochrome A, parental shading

As sessile organisms, it is crucial to sense their surroundings and mount appropriate responses to different environment. Shade avoidance syndrome (SAS) is a typical phenotypic plasticity, which is evolved to reach for more light upon exposure to shade (Casal, 2012). The core molecular mechanism of SAS is light signaling transduction. SAS affects many agronomic traits such as reduced grain yield or plant biomass (Tang and Liesche, 2017; Wille et al., 2017). So the understanding on SAS could contribute to basic research and agriculture application.

In past years, enormous progress has been made in molecular link from shade light perception to shade phenotypic architecture. Chinese researchers have done a lot of work in this field.

Arabidopsis FAR-RED ELONGATED HYPOCOTYL3 (FHY3) and FAR-RED IMPAIRED RESPONSE1 (FAR1), two homologous proteins, which have been reported to regulate the balance between growth and defense responses under shade condition (Liu et al., 2019), were shown to regulate the progression of leaf senescence in this Research Topic (Xie et al.). Xie et al. identified FHY3, PHYTOCHROME-INTERACTING FACTOR 5 (PIF5), and ETHYLENE-INSENSITIVE3 (EIN3) complex and explained how this complex regulates the expression of *ORE1* (NAC transcription factor). This study emphasized that the light and ethylene signaling coordinately regulate leaf senescence.

Despite our molecular understanding of shade-induced phenotypes, little is known about how this program is integrated with other environmental factors, such as nitrogen. Yang et al. found that both hypocotyl elongation and leaf expansion promoted by the shade treatment were reduced by the high-N treatment; high-N-induced leaf narrowing and thickening were reduced by the shade treatment, and they identified SHADE AVOIDANCE 3 (SAV3) as a master switch responsive to multiple environmental stimuli.

Soybean, as one of the economically important crops, displays obvious SAS. Mu et al. generated *GmBIC* overexpression lines and *Gmbic1a1b2a2b* quadruple mutant. The modified low blue light (LBL)-induced phenotypes of these genetic materials demonstrated the function of GmBICs (BIC:Blue-light Inhibitors of Cryptochromes) in SAS. This study provided essential genetic resources for breeding of shade-tolerant soybean cultivars in future.

One Mini Review in this Research Topic is focused on the role of phytochrome A (phyA) to hint the difference between shade avoidance and shade tolerance plants (Xu et al.). Although it is a mini review, it summarized almost all recently progress on phyA and its downstream components. Modifying the negative regulator of SAS may be a shortcut to obtain high-yield shade tolerance crops.

One opinion article is talking about parental shading on subsequent seed dormancy and germination control. This is a unique angle to consider the effects of exposure of the parental plants to shade on subsequent seed. It will enlarge our horizon on SAS.

With the increasing of population and a predicted decrease in total arable land, denser planting is required to supply the increasing food demand. SAS is one of the limiting issues for denser planting. So we hope more scientists care about this field and join in us.

## Author contributions

All authors listed have made a substantial, direct, and intellectual contribution to the work and approved it for publication.

## Conflict of interest

The authors declare that the research was conducted in the absence of any commercial or financial relationships that could be construed as a potential conflict of interest.

## Publisher's note

All claims expressed in this article are solely those of the authors and do not necessarily represent those of their affiliated organizations, or those of the publisher, the editors and the reviewers. Any product that may be evaluated in this article, or claim that may be made by its manufacturer, is not guaranteed or endorsed by the publisher.
